# INEX – A binary neuronal model with inhibitory and excitatory synapses

**DOI:** 10.1186/1471-2202-12-S1-P260

**Published:** 2011-07-18

**Authors:** Kerstin Lenk, Barbara Priwitzer

**Affiliations:** 1 Department of Information Technology/ Electronics/ Mechanical Engineering, Lausitz University of Applied Sciences, Senftenberg, 01968 Germany

## 

Our aim is to develop a simple model which is suitable to simulate concentration-response curves as observed in in-vitro experiments with multielectrode array (MEA) neurochips. In an in-vitro experiment approximately 10.000 neurons of the frontal cortex of embryonic mice [1] are cultivated on a MEA neurochip [2]. Neuro-active substances like bicuculline are added to the network. Based on the recorded data, various features [3] are calculated adapted from spikes and bursts. The features are separately displayed in concentration-response curves [4] which show the logarithm of the substance concentration and the chosen feature.

The developed INEX (inhibitory-excitatory) model is a cellular automaton whose cells are neurons with two possible states: ON or OFF. Each neuron obtains several inputs and produces exactly one output (respectively 0 or 1). Furthermore, it is phenomenological model where the neurons are described as black boxes. The probability if a spike occurs in time slice was calculated using a Poisson process [5]. Neurons are connected by either inhibitory or excitatory synapses with varying strength. The corresponding parameters are called weights. The network is fully connected and has direct feedbacks. Additionally, a spike time history was added. The aim was to vary the parameters of the model in such a way that we obtain a sigmoid concentration-response curve to simulate excitatory and inhibitory effects in neuronal networks.

**Figure 1:**
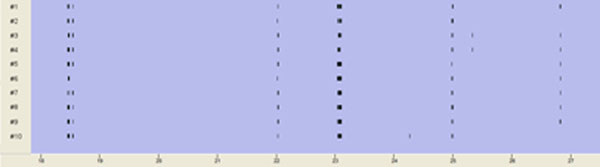
In each row, bursting spike trains of the first ten simulated neurons are displayed. Each dash marks a spike. The time scale (below) is in seconds.

A network with 100 neurons ran over 10 seconds with varying weights and Δt = 1 ms. Ninety inhibitory synapses with weights between -0.2 and 0 and ten excitatory synapses with weights between 0 and 0.7 are used. We detected spikes and bursts (figure 1) as known from experiments with MEA neurochips. Thereafter, the same network ran over 18 minutes. The excitatory weights are reduced in six steps respectively by 0.05 every 3 minutes. The mean spike rate for each step is calculated and displayed in a concentration-response curve [6].

The INEX model shows potential to simulate inhibitory and excitatory effects which are also observed in experiments with MEA neurochips. A sigmoid concentration-response curve can be obtained by the simulation. We will work on parallelisation of processes to decrease the run time of the algorithm.
